# Identification of the key immune gene NR3C1 as a diagnostic biomarker in differentiating ovarian borderline tumors from benign tumors

**DOI:** 10.3389/fcell.2025.1602192

**Published:** 2025-09-01

**Authors:** Shiying Chen, Yumin Ke, Yajing Xie, Zhimei Zhou, Weihong Chen, Li Huang, Liying Sheng, Yueli Wang, Shunlan Liu, Zhuna Wu

**Affiliations:** ^1^ Department of Gynecology and Obstetrics, The Second Affiliated Hospital of Fujian Medical University, Quanzhou, Fujian, China; ^2^ Department of Ultrasound, The Second Affiliated Hospital of Fujian Medical University, Quanzhou, Fujian, China

**Keywords:** machine-learning, borderline ovarian tumors(BOTs), benign ovarian tumors(BeOTs), NR3C1, immune-related biomarkers

## Abstract

**Background:**

This study aims to evaluate novel immune-related biomarkers for distinguishing borderline ovarian tumors (BOTs) from Benign ovarian tumors (BeOTs), addressing the diagnostic challenges posed by their intermediate biological behavior between benign and malignant neoplasms.

**Methods:**

We obtained the microarray expression profiles from the datasets (GSE4122 + GSE6822 + GSE36668) in the Gene Expression Omnibus (GEO) database and integrated them with the immune-related genes in the ImmPort database. Differentially immune-related genes (DIRGs) underwent Gene Ontology (GO) and Kyoto Encyclopedia of Genes and Genomes (KEGG) enrichment analysis. Protein-protein interaction (PPI) network was built to explore the connection. Candidate biomarkers were identified using the Least absolute shrinkage and selection operator (LASSO) and support vector machine-recursive feature elimination (SVM-RFE), with their diagnostic ability evaluated using Receiver operating characteristic (ROC) curves. A nomogram was constructed to predict BOTs. To validate the diagnostic potential and expression profiles, immunohistochemistry (IHC) analysis was performed in conjunction with the evaluation of an independent test group. We characterized the infiltration profiles of 22 immune cell types in BOTs through the CIBERSORT algorithm.

**Results:**

We identified 26 DIRGs between BOTs and BeOTs. These DIRGs were primarily associated with the positive regulation of transferase activity, the positive regulation of epithelial cell proliferation, and the positive regulation of the MAPK cascade. KEGG analysis indicated enrichment of Rap1 and PI3K-Akt signaling pathways. FGFR3, GNAI1, NR3C1, and PDGFA were found to have potential diagnostic value for BOTs (AUC_FGFR3_ = 0.883, AUC_GNAI1_ = 0.789, AUC_NR3C1_ = 0.760, AUC_PDGFA_ = 0.783) and further validated in the test group (AUC_FGFR3_ = 0.917, AUC_GNAI1_ = 0.900, AUC_NR3C1_ = 0.867, AUC_PDGFA_ = 0.833). Low expression of NR3C1 and GNAI1 and high expression of FGFR3 and PDGFA are associated with the development of BOTs. In addition, NR3C1 negatively correlated with CD4 memory resting T cells, as well as positively correlated with T cells gamma delta (P < 0.05).

**Conclusion:**

Our study findings suggested that NR3C1 may serve as an immune-related diagnostic biomarker for BOTs, offering a novel perspective for investigating the development and diagnosis of BOTs.

## Introduction

BOTs represent 10%–20% of epithelial ovarian tumors, constituting a distinct pathological entity with clinical and histological features intermediate between benign and malignant neoplasms. The lack of specific screening modalities and the absence of early clinical symptoms pose significant challenges to accurate diagnosis. Most patients are diagnosed incidentally during routine physical examinations or upon presentation with nonspecific abdominal symptoms such as pain or distension. Despite the majority of patients having a favorable prognosis and high overall survival rates, there has been an increasing incidence in recent years, with recurrence rates ranging from 5% to 34%. A subset of these cases demonstrates a propensity for malignant transformation, particularly those with high recurrence rates and seeding metastases, leading to less favorable outcomes (Vo et al., 2019; Sangnier et al., 2021; Raad et al., 2020; Armstrong et al., 2021).

The factors and molecular pathways implicated in the initiation and progression of BOTs are intricate and diverse, with ongoing discovery and exploration of diagnostic markers such as AGR2 (Armes et al., 2013), Rb/p105 (Masciullo et al., 2020), Aurora (Alkhateeb et al., 2021), among others. However, many of these markers have inherent limitations in terms of diagnostic sensitivity or specificity. Therefore, we hope to identify more efficient diagnostic biomarkers for BOTs through molecular biological information technology, enabling early diagnosis and treatment of BOTs, and reducing recurrence.

There is a growing body of research that establishes a connection between the immune microenvironment and the development and progression of cancer. Cancerous tumors must evade anti-tumor immune effects to grow gradually. The tumor immune microenvironment (TIME) consists of tumor cells, immune cells, and cytokines, and the interactions among these components determine the direction of anti-tumor immunity. Understanding the immunological characteristics of TIME has led to the development of new immunotherapeutic approaches and the identification of potentially useful biomarkers for clinical diagnosis, particularly for patients with challenging diseases, significantly improving their survival and prognosis (Gajewski et al., 2013; Lv et al., 2022). Therefore, this study aims to explore a highly efficient and specific method based on immune-related genes to assist in the clinical diagnosis and distinction of BOTs, thereby enhancing the prognosis and survival outcomes of BOTs patients.

## Methods

### Data collection and processing

We obtained the train group datasets (GSE4122+GSE6822+GSE36668) from the GEO database (https://www.ncbi.nlm.nih.gov/geo/), including 16 BOTs samples and 24 BeOTs samples. The test group dataset (GSE51088, including 12 BOTs and 5 BeOTs) (Table 1). Microarray probes were mapped to gene symbols in each dataset based on their probe annotation files. Genes detected by multiple probes were represented by aggregated mean expression values derived from corresponding probe measurements. To merge the three independent datasets into a unified meta-cohort, we implemented the “SVA” R package for batch effect correction (Supplementary Figure S1). The “limma” package in R was used for normalization of raw data and background correction (Ritchie et al., 2015). Differentially expressed genes (DEGs) were defined as those genes meeting the threshold of |log2 fold change (FC)| > 1 with an adjusted p-value < 0.05. The Immune-related genes (IRGs) Data were downloaded from the ImmPort database (https://www.immport.org/shared/) (Supplementary Table S1). Subsequently, DIRGs were identified as the intersection of IRGs and DEGs of BOTs.

**TABLE 1:** GEO database data of BOTs mRNA expression profile.

Dataset ID	Platform	BOTs	BeOTs
Train Group			
GSE4122	GPL201-30390	3	18
GSE6822	GPL80-30376	9	6
GSE36668	GPL570-55999	4	0
		16	24
Test Group			
GSE51088	GPL7264-9589	12	5

### Function and pathway enrichment of DIRGs

Functional annotation analysis was conducted through GO and KEGG pathway enrichment using the R packages ‘clusterProfiler’ (Yu et al., 2012), ‘org.Hs.e.g.,.db’, ‘enrichplot’, and ‘DOSE’. We performed the GO enrichment analysis using the full set of IRGs as the background gene set. Enriched terms were systematically characterized across three GO domains: biological processes (BP), molecular functions (MF), and cellular components (CC), along with curated KEGG signaling pathways. Enrichment results were visualized through dot plot representations generated by the R package “ggplot2”, employing a modular visualization framework. Statistical significance for functional enrichment was established using a p-value threshold of < 0.05.

### PPI network construction and analysis

We employed the STRING website (https://string-db.org/) (von Mering et al., 2003) to search for a PPI network using 26 DIRGs in the “multiple proteins” module and “*Homo sapiens*” in the organism module. Based on the protein IDs, corresponding gene symbols were assigned using the STRING Database. PPIs were filtered using a high confidence interaction score threshold (≥ 0.700), and those lacking associated gene symbols were excluded. Subsequently, the calculation of MCC was performed using the [cytoHubba] plugin (version 0.1) of Cytoscape software (version 3.10.0) (https://cytoscape.org/) (Chin et al., 2014) to identify key hub genes with degrees ranking in the top 10 based on the topology of protein-protein interaction networks. This algorithm calculates the connection pattern score of each node in the network to construct the PPI network.

### Building a model to predict BOT diagnosis based on DIRGs

Significantly diagnostic biomarkers were identified through the selection of DIRGs via Spearman correlation analysis in BOTs with cutoff = 0.3, pFilter = 0.05. We identified these biomarkers by combining the LASSO algorithm with the mSVM-RFE method. To perform LASSO, an algorithm used in regression analysis for variable selection to prevent overfitting, we utilized the “glmnet” package (Friedman et al., 2010). Meanwhile, the mSVM-RFE algorithm was executed with the help of the “e1071″R package (Zhou and Tuck, 2007). This algorithm makes use of resampling methods in each iteration. Its purpose is to stabilize the rankings of features and to pinpoint the most relevant features. It does so by eliminating the feature vectors produced by the SVM through supervised machine-learning techniques. Using the R package “pROC” to conduct ROC curves on the dataset. The area under the curve (AUC) value was calculated to assess the ability of the expected biomarker to distinguish BOTs from BeOTs tissue.

### Establish and validate the PCA and nomogram model for the diagnostic ability of BOTs

PCA reduces the dimensionality of gene expression data through eigenvalue decomposition and quantifies the statistical significance of differences between groups based on grouped confidence ellipses. PCA was used to further verify the diagnostic ability of DEGs for BOTs using the “limma” and “ggplot2” R packages. A nomogram model was constructed using the “rms” and “rmda” packages to predict the diagnosis of BOTs. Each factor’s score is represented as “points,” while the cumulative score of all factors is referred to as “total points.” Subsequently, calibration curves were generated to assess the predictive performance of the nomogram model.

### Evaluation of immune cell infiltration in BOTs and biomarkers

We utilized the CiberSort algorithm (http://cibersort.stanford.edu/) to quantify the proportion of 22 infiltration immune cells in BOTs (Gao et al., 2020) and conducted the penetration difference between the two groups using the “corrplot”, “reshape2”, and “ggpubr” packages in R software. Spearman’s correlation analysis was employed to investigate the relationship between screened diagnostic biomarkers and the levels of immune-infiltrating cells, and the results were visualized using the “ggplot2″ R package.

### Patient and tissue samples

We collected 40 paraffin-embedded specimens from patients with BOTs and 43 with BeOTs who underwent ovarian cystectomy or oophorectomy at the Second Affiliated Hospital of Fujian Medical University between January 2019 and May 2024. This study was approved by the Research Ethics Committee of the Second Affiliated Hospital of Fujian Medical University before commencement.

### Immunohistochemistry (IHC)

The IHC staining method using an anti-NR3C1 antibody was employed to categorize the intensity ratio of staining in specimens. The scoring criteria were as follows: Based on the proportion of positive cells among all tissue cells and the intensity of positive cell staining, the experimental results were determined as follows: (A) Score based on the number of stained cells. If the number of positive cells is less than 1/3 of the total number of cells, it is scored as 1 point; if it is between 1/3 and 2/3, it is scored as 2 points; if it is greater than or equal to 2/3, it is scored as 3 points. (B) Score based on the intensity of staining. If the staining is negative, it is scored as 0 points; if the staining is light yellow, it is scored as 1 point; if the staining is brownish yellow, it is scored as 2 points; if it is tan, it is scored as 3 points. The total score = A x B. Subsequently, the slide samples were divided into the low-expression and the high-expression groups, which were defined by total scores <6 and ≥ 6, respectively. The pathological diagnosis for tissue samples from study patients was conducted by two gynecologic oncology pathologists.

### Quantitative real-time PCR (qRT-PCR)

We extracted total RNA from frozen ovarian tissues after oophorectomy using TRIzol reagent (Beyotime, Biotechnology, China). Subsequently, we synthesized cDNA following the manufacturer’s instructions (TaKaRa, Japan). We employed GAPDH as an internal reference gene and calculated the relative mRNA expression levels of NR3C1 using the 2^−ΔΔCT^ method. We performed qRT-PCR in triplicate for each sample across three independent experimental replicates.

### Statistical methods

All statistical analyses were conducted using R software (v.4.1.1). The Mann-Whitney U test was employed to compare different groups, and the Chi-square test was utilized for the comparison of a 2 × 2 contingency table. The analyses included LASSO regression, SVM-RFE algorithm, ROC analysis, Spearman’s correlation, and unpaired t-test. Statistical significance was defined as P < 0.05.

## Results

### Study procedure

**FIGURE 1:**
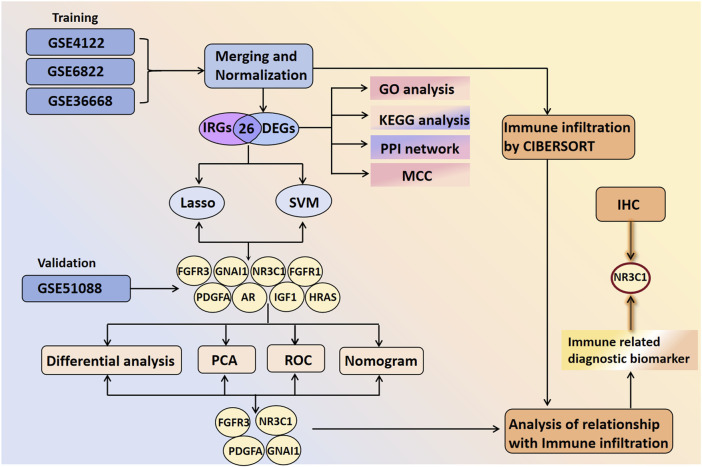
The flowchart of the overall study.

Figure 1 shows the analytical procedure outlined in the study. Microarray data were obtained from the GEO database. Based on the probe annotation file, the microarray probes were associated with the gene symbols in each dataset. DEGs were intersected with IRGs to obtain DIRGs. Enrichment analysis of DIRGs was performed using GO and KEGG databases. Candidate overlapping genes were further screened through PPI networks as well as two machine learning algorithms (LASSO and SVM-RFE). The predictive ability of the biomarkers was validated using principal component analysis (PCA) and ROC curves, which were further verified in the GSE51088 dataset. The composition pattern of 22 immune cells in BOTs was calculated using the Cibersort algorithm, followed by an analysis of immune cell correlations with diagnostic biomarkers. Finally, IHC staining was conducted on paraffin-embedded specimens meeting the inclusion criteria to validate our findings.

### Identification of DIRGs in BOTs

**FIGURE 2:**
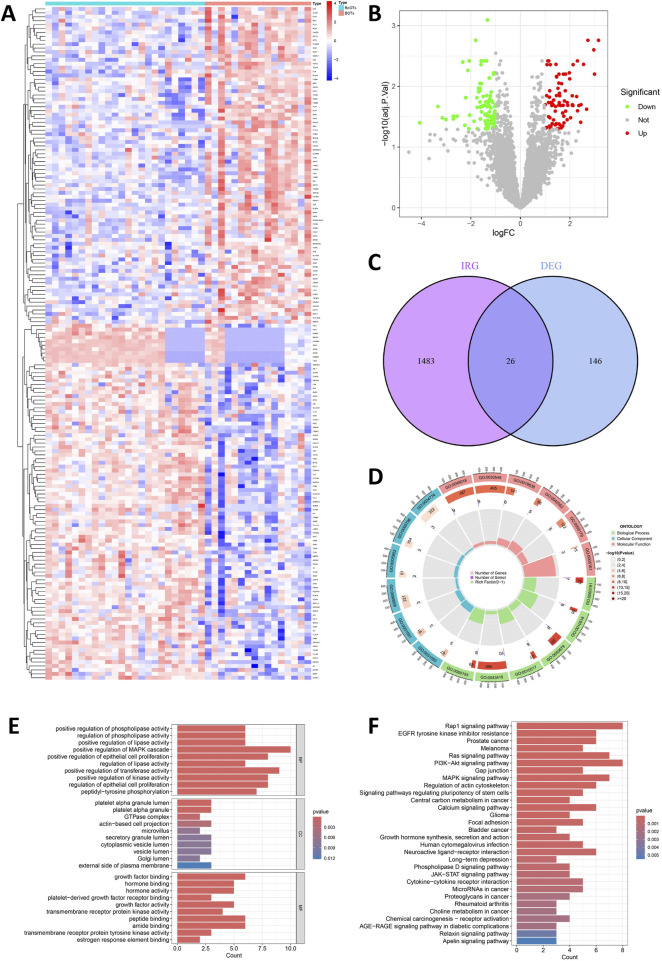
Detection and function of DIRGs between BOTs and BeOTs. **(A)** Heatmap showing the expression levels of top differentially expressed genes (DEGs) between BeOTs and BOTs groups. Each row represents a gene (with gene names shown in row annotations), and each column represents a sample (sample IDs not shown). The color scale represents row Z-score normalized expression values, with blue indicating low expression and red indicating high expression. Sample groups are annotated at the top (BeOTs vs. BOTs). **(B)** Volcano plot shows the distribution of DEGs. The x-axis shows log2 fold change (logFC), and the y-axis represents -log10 (adjusted P-value). Red dots denote significantly upregulated genes (adjusted P < 0.05, positive logFC) with higher expression in BOTs. Green dots denote significantly downregulated genes (adjusted P < 0.05, negative logFC) with lower expression in BOTs. Gray dots represent non-significant genes. Vertical dashed lines indicate |logFC| > 1 thresholds; horizontal dashed line marks the significance threshold (adjusted P = 0.05). **(C)** A Venn plot was used to identify the intersection of DIRGs between the IRGs and the DEGs. **(D)** Circular visualization of GO terms. Enriched terms across BP (outer sector), CC (middle sector), and MF (inner sector) are shown. Bar height: Gene count (0–400). Bar color: Significance (-log_10_P-value; darker = better). Inner marks: Rich Factor (0–1; specificity). Labels: GO term IDs. **(E)** Bar plot of enriched Gene Ontology (GO) terms. Top 10 significantly enriched terms (p < 0.05) are shown for each ontology: Biological Process (top), Cellular Component (middle), and Molecular Function (bottom). The x-axis indicates the Gene Ratio (number of enriched genes in term÷total background genes). Terms are ranked by statistical significance. **(F)** Bar plot of enriched KEGG pathways. Top 30 significantly enriched pathways (p < 0.05). The x-axis indicates the Gene Ratio (number of enriched genes in pathway÷total background genes). Pathways are ranked by statistical significance.

From three integrated datasets (GSE4122, GSE6822, and GSE36668), we identified 172 DEGs between 16 BOT patients and 24 BeOTs samples. These DEGs were filtered using thresholds of adj.P.Val < 0.05 and |log2FC| > 1 (Figure 2A; Supplementary Table S2). A total of 91 genes demonstrated significant downregulation, while a lower number (81 genes) showed significant upregulation (Figure 2B). IRGs were refined to 1,509 high-confidence candidates through standardization of gene names (trimming spaces) and deduplication before intersection analysis (Supplementary Table S3). To identify DIRGs in BOTs, we performed an intersection of the DEGs and IRGs to identify 26 DIRGs (Figure 2C; Supplementary Table S4). Among these DIRGs, 16 DIRGs were downregulated, whereas 10 DIRGs were significantly upregulated in the BOTs.

### Functional enrichment analysis

Using the “ClusterProfiler” package in R, functional enrichment analysis was carried out to investigate the potential biological functions and signaling pathways associated with the 26 DIRGs. These biological processes (BP) were mainly associated with positive regulation of the MAPK cascade, epithelial cell proliferation, and positive regulation of transferase activity. The cellular components (CC) of the DIRGs are primarily focused on the platelet alpha granule lumen, actin-based cell projection, and vesicle lumen. The molecular function (MF) of the DIRGs was significantly enriched in receptor-ligand activity, signal receptor activator activity, and growth factor binding (P < 0.05, Figures 2D,E, Supplementary S1). Furthermore, KEGG enrichment results highlighted the 26 DIRGs’ major involvement in several signaling pathways, including Rap1, PI3K-Akt, Ras, and MAPK signaling pathways. These findings will facilitate further exploration into potential mechanisms underlying BOT development (P < 0.05, Figure 2F, Supplementary S2).

### PPI network construction and hub gene selection

**FIGURE 3:**
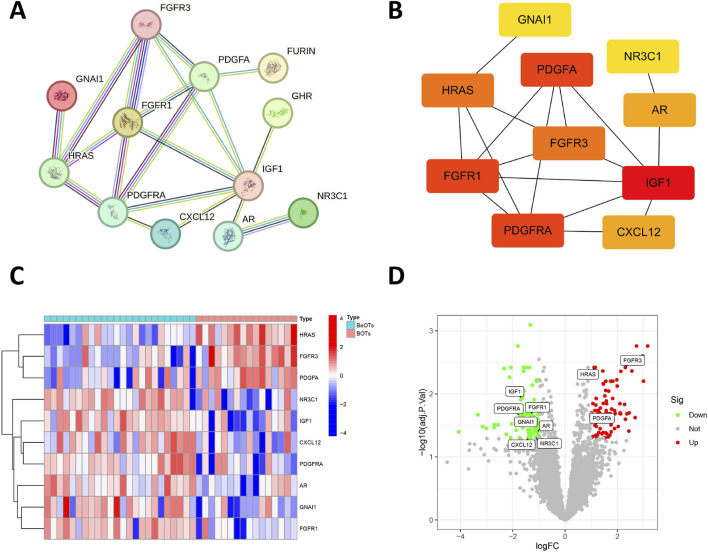
Association between DIRGs and hub genes. **(A)** PPI network of the 12 DIRGs constructed using the STRING database. Nodes represent proteins (gene products) with colors indicating their functional clustering: proteins with similar biological roles share the same color. Lines (edges) represent predicted functional associations with colors denoting the types of supporting evidence: experimentally validated interactions (pink), database-curated interactions (blue), co-expression evidence (black), and text mining predictions (yellow). Line thickness reflects the confidence score of each interaction. **(B)** Top 10 hub genes identified by the MCC algorithm using the cytoHubba plugin in Cytoscape. Node color intensity reflects MCC score, where warmer (redder) colors indicate higher centrality scores and greater importance in the network. **(C)** Heatmap showing expression patterns of the top 10 hub DIRGs between BeOTs and BOTs groups. The color gradient represents row Z-score normalized expression values, with blue indicating low expression (negative Z-score) and red indicating high expression (positive Z-score). Each row represents a gene (labeled on the right), and each column represents a sample. Sample groups are annotated at the top (BeOTs vs. BOTs). **(D)** volcano plot presenting the top 10 hub DIRGs.

To construct a PPI network, we employed the STRING database (https://string-db.org/) by inputting 26 DIRGs into the “multiple proteins” module and selecting *H. sapiens* as the target species. Following the removal of disconnected nodes, the PPI network retained 12 interconnected DIRGs (Figure 3A). Subsequently, cluster analysis of the network genes was performed using the cytoHubba plugin in Cytoscape software. 10 hub nodes prioritized by the MCC (Maximal Clique Centrality) algorithm were identified and categorized (Figure 3B). The expression levels of FGFR3, HRAS, and PDGFA genes were observed to be upregulated, whereas the expression levels of GNAI1, PDGFRA, CXCL12, AR, NR3C1, FGFR1, and IGF1 genes were found to be downregulated (Figures 3C,D).

### Correlation of prospective biomarkers in BOTs

**FIGURE 4:**
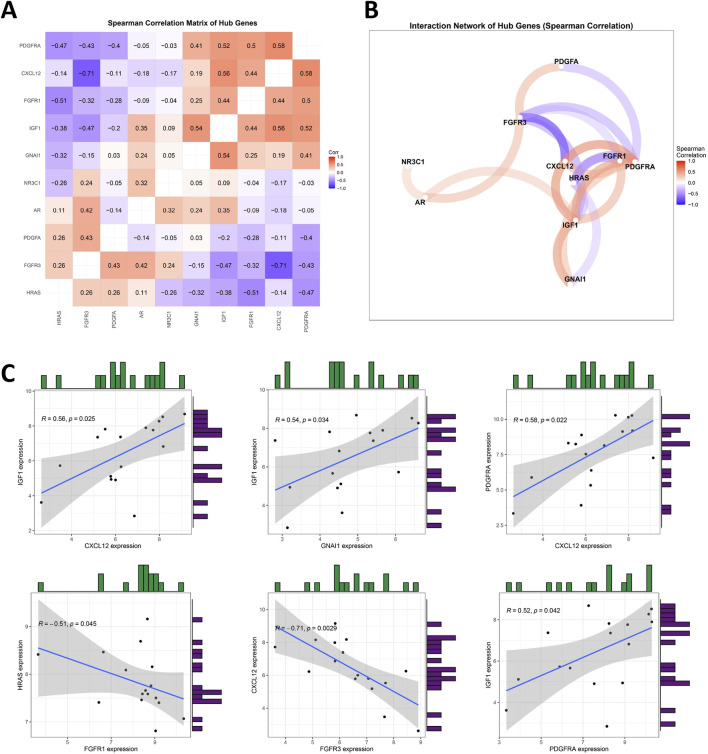
Analyzing the Correlation among DIRGs. **(A)** This heatmap visualizes pairwise Spearman correlations between 10 hub genes. Hierarchical clustering reorders genes to group similar correlation patterns. Red indicates positive correlations (max ρ = 0.58), blue indicates negative correlations (min ρ = −0.71), and white denotes weak/no correlation. Absolute correlations below the cutoff (|ρ| < 0.3) are filtered out. Numerical values represent correlation coefficients (ρ). Generated using “ggcorrplot” in R. **(B)** Nodes represent hub genes; edges (colored lines) indicate statistically significant correlations (|ρ| > 0.3). Edge colors reflect correlation direction: red for positive correlations, blue for negative correlations. Node size and label font were enlarged for visibility. Generated using corrrnetwork_plot in R with a gradient legend (ρ: −1 to 1). **(C)** A scatter plot for some highly correlated DIRGs is provided. Positive correlation in red and negative correlation in blue. The darker the color, the greater the correlation.

To investigate the interrelationships among the 10 DIRGs, Spearman correlation analysis was conducted with a cutoff of 0.3, pFilter = 0.05. Leveraging the ‘tidyverse’ and ‘corrr’ packages in R, co-expression correlation heatmap (Figure 4A) and co-expression network diagram (Figure 4B) present key interactions included strong positive correlations between CXCL12 and PDGFRA (r = 0.58, p = 0.0194), and significant negative correlations between FGFR3 and CXCL12 (r = −0.71, p = 0.002) (Supplementary Tables S5, S6). Scatter plots showed that the expression level of IGF1 was significantly positively correlated with the levels of CXCL12, GNAI1, and PDGFRA. Furthermore, the expression level of FGFR3 was negatively correlated with CXCL12 expression (Figure 4C).

### Construction of a diagnostic model for BOTs

**FIGURE 5:**
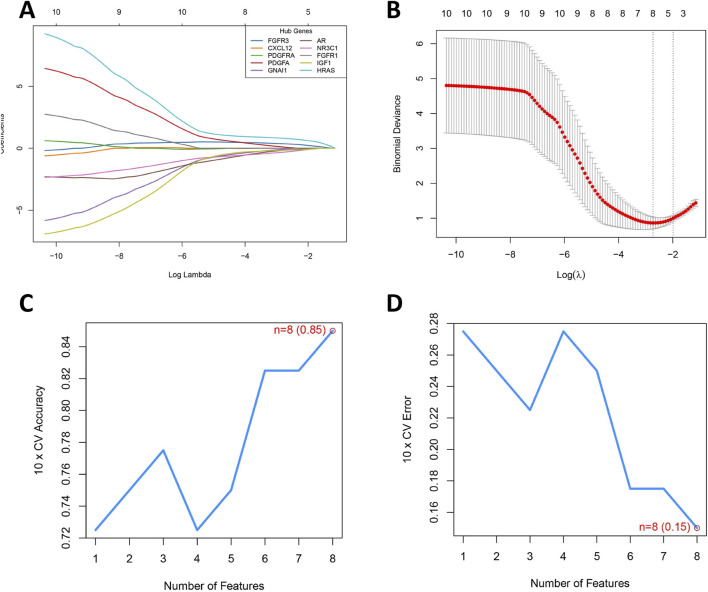
Development of a Prediction Model for BOTs. **(A)** The LASSO regression coefficient profiles of the 8 DIRGs are presented as a curve; each curve represents a gene’s coefficient path across regularization parameters (logλ). Gene-specific colors shown in legend (top-right). **(B)** The LASSO Cox regression model was utilized to generate a plot of partial likelihood deviance against log(λ). The left dotted line is at lambda. Min (at the minimum deviation), and the right dotted line is at lambda. 1se. **(C)** When n = 8, the curve of the total within sum of squared error curve under the corresponding cluster number n arrives at the “elbow point”. **(D)** Cross-validation error rate decreases as feature count increases, reaching its minimum (0.15) at 8 features (red point). The blue curve tracks mean error across 10-fold cross-validation, with the dashed line indicating chance-level performance (0.5).

To precisely identify key diagnostic biomarkers in BOTs, we used the Lasso algorithms (Figures 5A,B) and SVM-RFE (Figures 5C,D) algorithms to identify potential diagnostic biomarkers. It is worth noting that the DIRGs identified by the two algorithms were completely consistent. In the end, we provided a comprehensive summary of the 8 candidate genes (PDGFA, HRAS, GNAI1, NR3C1, FGFR3, IGF1, AR, and FGFR1) parameters and results from both LASSO and SVM-RFE algorithms, as shown in Table 2.

**TABLE 2:** Gene selection parameters and results from LASSO and SVM-RFE.

Method	Key parameters	Selected genes
LASSO	- Family: Binomial - α: 1 (L1 penalty) - λ: lambda.min - nfolds: 10 -Type.measure: Devian	FGFR3, PDGFA, GNAI1, AR, NR3C1, FGFR1, IGF1, HRAS
SVM-REF	- Kernel: Radial - Step size (k): 10 - halve. above: 50 - nfold: 10	PDGFA, HRAS, GNAI1, NR3C1, FGFR3, IGF1, AR, FGFR1

### Comprehensive characterization and verification of the eight key DIRGs

**FIGURE 6:**
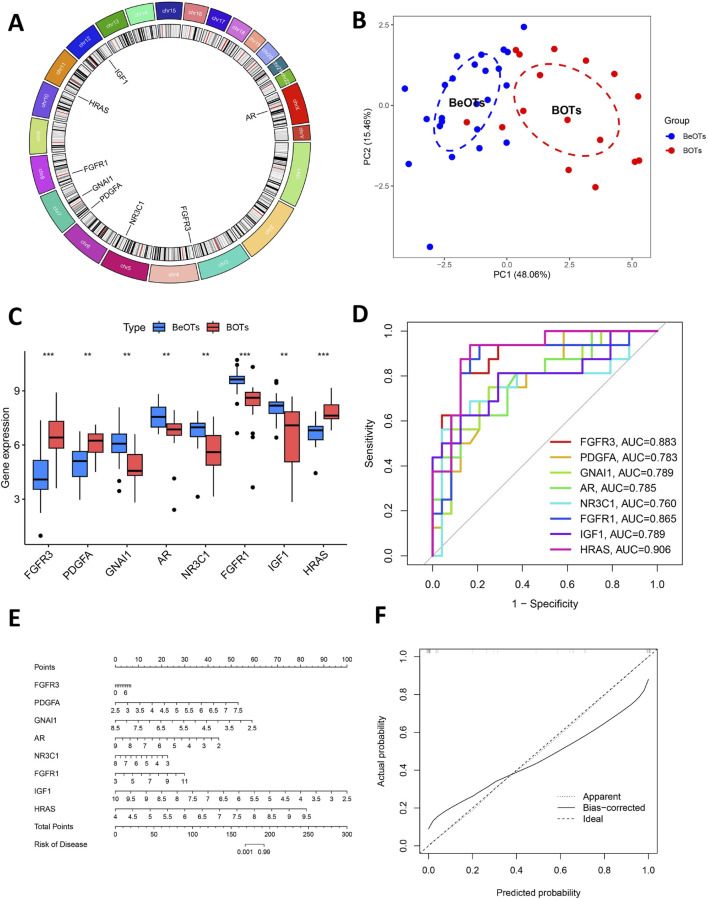
Additional analysis of eight key DIRGs. **(A)** The positions on the chromosome of the right crucial DIRGs. **(B)** The PCA plot displays the sample distribution based on the expression profiles of 8 key DIRGs. Samples are colored by group (Control = blue, Treat = red), with dashed ellipses representing 95% confidence intervals for each group. The x- and y-axes correspond to the first two principal components (PC1 and PC2), with percentage values of explained variance indicated in parentheses. **(C)** The comparative expression levels of three crucial DIRGs in BOTs as opposed to BeOTs are presented by the train group datasets (GSE4122+GSE6822+GSE36668). **(D)** The efficacies of three crucial DIRGs in predicting BOTs within the train group were validated by ROC curves. **(E)** Nomogram for predicting the risk of borderline ovarian tumor based on eight differentially immune-related genes (DIRGs). The nomogram includes eight genes (FGFR3, PDGFA, GNAI1, AR, NR3C1, FGFR1, IGF1, and HRAS). For each gene, a point is assigned according to its expression level. The total points are calculated by summing the points of all genes and then converted to the risk of disease. **(F)** Calibration curve of the nomogram. The calibration curve compares the predicted risk (x-axis) with the actual observed risk (y-axis). The diagonal line represents the ideal prediction. The solid red line (Apparent) and the dashed blue line (Bias-corrected) show the performance of the model before and after bias correction by bootstrapping (1,000 repetitions), respectively.

**FIGURE 7:**
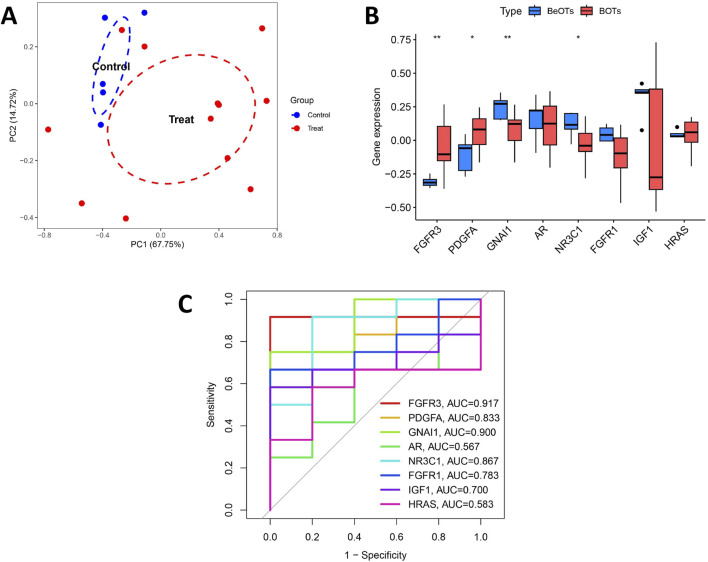
Validation of diagnostic biomarkers in the test group. **(A)** The PCA plot displays the sample distribution based on the expression profiles of 8 key DIRGs in the test dataset. Samples are colored by group (Control = blue, Treat = red). The x- and y-axes correspond to the first two principal components (PC1 and PC2), with percentage values of explained variance indicated in parentheses. **(B)** Boxplot showed the expression of DIRGs between BOTs and BeOTs in the test group (GSE51088) (*P < 0.05, **P < 0.01). **(C)** The ROC curve of the diagnostic efficacy verification of eight immune-related hug genes in the test group.

Figure 6A shows the location of 8 DIRGs on the chromosome. PCA results showed that the first two principal components (PC1 and PC2), with percentage values of explained variance indicated in parentheses (“PC1 = 48.06%; PC2 = 15.46%”). The result demonstrated that these 8 candidate genes possessed robust discriminatory capacity between BOTs and BeOTs, suggesting their critical roles in BOT diagnosis (Figure 6B; Supplementary Table S7). As previously demonstrated (via heatmap) and differential expression analysis (Figure 6C), the 8 DIRGs, including five downregulated genes (GNAI1, AR, NR3C1, FGFR1, and IGF1) and three upregulated genes (FGFR3, PDGFA, and HRAS) in BOTs, showed significant expression differences between the two groups. ROC analyses were performed on potential DIRGs to assess their predictive accuracy. The results revealed that the AUC values of the 8 DIRGs all exceeded 0.7, indicating a good discriminatory diagnostic value for these 8 biomarkers in BOTs (Figure 6D). To provide a quantitative tool for clinical application, we developed a nomogram that incorporates the eight DIRGs (Figure 6E). Each gene expression level is converted to a point score, and the total points are used to predict the risk of borderline ovarian tumor. The nomogram demonstrated good discriminative ability with a C-index of value. Furthermore, the calibration curve (Figure 6F) showed good agreement between the predicted risk and the actual outcome, especially after bias correction. Furthermore, to select more reliable and accurate DIRGs, we conducted validation of the expression levels of 8 DEGs using the GSE51088 dataset. The PCA results showed that the first two principal components (PC1 and PC2), with percentage values of explained variance indicated in parentheses (“PC1 = 67.75%; PC2 = 14.72%”). The plot visualizes sample distribution based on the expression patterns of the 8 DIRGs (Figure 7A; Supplementary Table S8). The findings revealed that FGFR3 and PDGFA exhibited significantly higher expression levels, while NR3C1 and GNAI1 showed significantly lower expression levels in BOTs tissue (Figure 7B). The AUC values of the ROC curves for these four DIRGs (AUC_FGFR3_ = 0.917; AUC_PDGFA_ = 0.833; AUC_NR3C1_ = 0.867; AUC_GNAI1_ = 0.900) demonstrated a superior predictive power for BOTs compared to BeOTs (Figure 7C).

### NR3C1 is associated with immune infiltration

**FIGURE 8:**
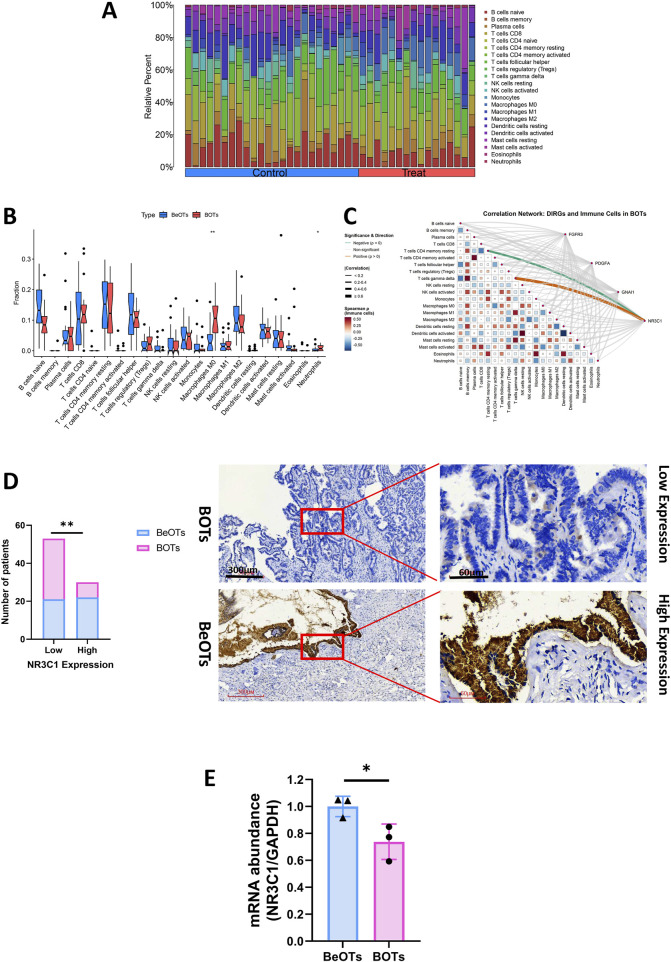
The evaluation of four DIRGs related to immune infiltration. **(A)** Stacked bar plot showing the relative abundance of 22 immune cell subtype proportions between BOTs and BeOTs samples. **(B)** Differential immune cell infiltration profiles between BOTs and BeOTs. A stacked bar plot displays relative proportions of 22 immune cell types in each sample. Samples are grouped by tumor type (left: BeOTs, n = BeOTs number; right: BOTs, n = BOTs number). Color-coded segments indicate the relative abundance of specific immune cell subsets. **(C)** DIRGs-immune cell correlation network diagram: It shows the interactions between four genes (FGFR3, PDGFA, GNA11, NR3C1) and 22 types of immune cells in BOTs. Line colors indicate the direction of correlation (orange: positive correlation; green: negative correlation; gray: non-significant). Line thickness indicates the strength (thin → thick: weak → strong). The heatmap at the bottom shows the interaction relationships between immune cells (red: positive correlation; blue: negative correlation). All significant lines have passed statistical tests (p < 0.05). **(D)** Distribution of patients with 40 BOTs and 43 BeOTs stratified by NR3C1 expression levels. The bar plot illustrates the number of patients with BOTs and BeOTs categorized according to NR3C1 expression levels (Low vs High; y-axis: Number of patients). Statistical analysis compared distribution differences between BOTs and BeOTs within each NR3C1 expression group using the Chi-square test. Significance level was set at p < 0.05, with ** denoting p < 0.01. Representative images (×40 and ×200) of IHC staining for NR3C1 in BOTs and BeOTs specimens (high expression versus low expression). **(E)** qRT-PCR of NR3C1 expression in BOTs when compared with BeOTs specimens. Scale bars are shown. *p < 0.05. p-values were calculated by chi-square tests.

To gain a more intensive understanding of the association between immune cell infiltration and BOTs, we utilized the CIBERSORT algorithm to determine the relative abundances of 22 types of immune cells in both the control and BOT samples (Figure 8A). Subsequently, we made a comparison of immune cell infiltration in BOT samples and BeOTs samples. The findings revealed a significantly higher abundance of Macrophages M0 and Neutrophils in the BOTs group (Figure 8B). Furthermore, we investigated the association between key diagnostic biomarkers and distinct infiltrating immune cells. Our correlation analysis revealed a statistically significant positive association between NR3C1 expression and T cells gamma delta infiltration (Spearman r = 0.664, p = 0.005), suggesting a potential role for NR3C1 in modulating this immune subset. Conversely, NR3C1 showed a significant negative correlation with T cells CD4 memory resting levels (r = −0.524, p = 0.040), indicating an inverse relationship with this cell population. However, no significant correlation was observed between the expression of FGFR3, GNAI1, and PDGFA genes and immune cells (Figure 8C) (Supplementary Table S9). These observations provide support for the close relationship between NR3C1 and immune activity, highlighting its potential significance in regulating immune cell function in BOTs. We further evaluated the expression of NR3C1 in BOTs and BeOTs tissues by IHC and found that the low expression of NR3C1 was significantly correlated with BOTs (Figure 8D, P = 0.0031). We assessed the expression of NR3C1 using qRT-PCR and revealed that low expression of NR3C1 was associated with BOTs (Figure 8E; p < 0.05). It was further verified that NR3C1 has a high diagnostic ability in differentiating the two ovarian tumors.

## Discussion

BOTs are ovarian tumors that exhibit intermediate growth patterns and cytological features between benign and malignant tumors, with non-destructive stromal infiltration characteristics (Niu et al., 2021). Therefore, preoperative imaging examination and tumor marker detection often cannot provide a definitive diagnosis of BOTs, and the concordance rate of intraoperative frozen sections and postoperative pathology is also low. Some patients may require a second operation. Considering that there are currently no predictive indicators that can accurately distinguish BOTs from BeOTs, we conducted the above study to identify potential biomarkers for the diagnosis of BOTs, intending to provide some assistance for the clinical diagnosis and distinction of BOTs to improve the prognosis and survival outcomes of BOT patients.

With the advancement of bioinformatics technology, an increasing number of tumor diagnostic and therapeutic targets have been identified from a vast database of sample data. Concurrently, as more studies focus on the correlation between immune cell infiltration and the onset, progression, and prognosis of various diseases, the presence of immune targets becomes crucial for the outcome of disease immunotherapy. By conducting cross-analysis using IRGs data and DEGs, we identified 26 overlapping DIRGs. GO and KEGG analyses revealed that these DIRGs were predominantly enriched in MAPK cascade, Rap1, PI3K-Akt, and Ras signaling pathways. They exhibited receptor activator activity related to signaling pathways, cytokine activity regulation, and positive effects on epithelial cell proliferation. Notably, activation of MAPK subfamily members such as ERK, P38, and JUN can collaborate with NF-κB and interferon regulatory factor transcription factors to induce the expression of multiple genes while jointly regulating immune and inflammatory responses (Arthur and Ley, 2013). The PI3K-Akt signaling pathway is believed to participate in immune suppression within the ovarian tumor microenvironment by regulating tumor-associated macrophage expression (Cannon et al., 2015). These findings will contribute to a deeper understanding of immune regulation mechanisms associated with BOTs.

Our study employed two machine learning algorithms to validate 8 DIRGs and utilized the GSE51088 dataset for validation, confirming that NR3C1, GNAI1, FGFR3, and PDGFA are potential biomarkers for BOT diagnosis. Furthermore, to gain a deeper understanding of the tumor immune microenvironment, CiberSort analysis revealed an association between increased levels of Macrophages M0 and Neutrophils with the development of BOTs. Consistent with this finding, Martin J. Cannon et al. also demonstrated that ovarian tumor-associated macrophages may play a pivotal role in creating an immunosuppressive environment that hinders anti-tumor immune responses and promotes disease progression (Cannon et al., 2015). Ke Huang et al. proposed a method of integrating inflammation biomarkers and tumor biomarkers to enhance the diagnosis of borderline and malignant epithelial ovarian tumors. The study observed a gradual increase in inflammatory markers such as neutrophils, neutrophil-to-lymphocyte ratio (NLR), platelet-to-lymphocyte ratio (PLR), and monocyte-to-lymphocyte ratio (MLR) from benign to borderline and malignant ovarian tissues, which were significantly correlated with tumor progression and prognosis, consistent with our research findings. Furthermore, the study assessed the diagnostic system combining CA125 with NLR and PLR by constructing a multivariate Logistic regression model and determining optimal cutoff values for each indicator. The results indicated suggested cutoff values for CA125, NLR, and PLR to differentiate between benign and borderline epithelial ovarian tumors as 18.72 U/ml, 1.244, and 89, respectively, with sensitivities of 53.97%, 87.5%, and 92.06%, along with specificities of 100%, 69.87%, and 75.48%. This diagnostic system demonstrates greater accuracy in distinguishing the nature of epithelial ovarian tumors (EOTs) compared to single or arbitrary dual combinations, particularly for BOTs (Huang et al., 2023). Examination of immune infiltration in BOTs will facilitate a more comprehensive understanding of the cellular composition involved in immune suppression, thus offering new insights for innovative immune-boosting therapies.

Furthermore, we have identified diagnostic biomarkers associated with infiltrating immune cells. Our results indicate that NR3C1 may impact the development of BOTs by regulating the expression of T cells, gamma delta, and CD4 memory resting. This finding supports the immunoregulatory activity of NR3C1. The NR3C1 gene encodes the glucocorticoid receptor (GR), which specifically binds to promoter regions of glucocorticoid response genes in the cell nucleus to promote gene transcription. It also participates in cellular inflammatory responses and interferes with the activities of other immune-related transcription factors such as nuclear factor kappa B, activator of T cells nuclear factor, activator protein 1, interferon regulatory factor 3, cAMP response element binding protein, T-box transcription factor 21, GATA binding protein 3, etc., exerting anti-transcriptional inhibition (Xie et al., 2021). The GR signal is believed to play a role in the pathogenesis of diseases such as breast cancer, prostate cancer, and hematological tumors. There have been several studies investigating the association between NR3C1 and solid tumor diagnosis and treatment, both domestically and internationally. It has been demonstrated that abnormal expression of NR3C1 in various malignant tumors, including colon cancer, renal clear cell carcinoma, breast cancer, and pancreatic cancer, affects the proliferation and migration of cancer cells (Zhai et al., 2024; Yan et al., 2023; Dwyer et al., 2023; Wang et al., 2023), although the number of related studies remains relatively limited. Peng Zhai et al. study demonstrates that DNA methyltransferase 1-mediated NR3C1 DNA methylation can upregulate the transcription of CX40, thereby promoting angiogenesis in colorectal cancer. Restoration of NR3C1 suppresses the angiogenic, proliferative, survival, and oncogenic activities of colorectal cancer cells (Zhai et al., 2024). Yan, M. et al. demonstrated that knockout of the NR3C1 gene activates endoplasmic reticulum stress and affects cell division and migration of renal clear cell carcinoma through the ATF6-PINK1/BNIP3 pathway, providing a novel biological target for clinical treatment of chronic renal cell carcinoma (Yan et al., 2023). It has been confirmed that NR3C1 in pancreatic cancer is highly regulated by upstream miR-1270, and silencing NR3C1 can inhibit the malignant phenotype of pancreatic cancer cells (Wang et al., 2023). Fahai Chen et al. identified 5 immune-related hub genes associated with poor prognosis and response to trastuzumab treatment in breast cancer patients, with NR3C1 being one of them (Chen and Fang, 2022). Jin, X. et al. validated the potential diagnostic biomarker value of NR3C1 as a breast cancer marker using bioinformatics methods (Jin et al., 2023). Additionally, Junfeng Chen et al. established a risk model based on the four hypoxia-related genes NR3C1, ANXA2, AKAP12, and GPI to predict the prognosis and survival rate of endometrial cancer patients (Chen et al., 2022). These related research conclusions, to some extent, support the potential of NR3C1 as an immune-related diagnostic marker for solid malignant tumors.

After conducting a comprehensive literature review, several studies have been conducted to explore the correlation between NR3C1 and ovarian reproductive function and ovulation. Julian T. Pontes et al. examined the expression levels and immunohistochemical localization of NR3C1 in primordial and follicular ovarian tissues, revealing its presence in all types of ovarian tissues except for granulosa cells in primordial follicles, with varying intensity and location of expression. Additionally, elevated cortisol levels were found to be detrimental to follicle survival, leading to a significant reduction in the percentage of normal follicles (Pontes et al., 2019). Shumail Syed et al. identified two intronic variants (rs10482672 and rs11749561) within the NR3C1 gene that are linked to PCOS risk by regulating the stress response, marking the first study to designate NR3C1 as a risk gene for PCOS (Syed and Gragnoli, 2024). However, there is still a scarcity of clinical studies on NR3C1 concerning ovarian solid tumors, particularly those associated with BOTs, which have not been previously reported. Jennifer T. Veneris et al. investigated the association between NR3C1 and ovarian serous carcinoma through analysis of whole-genome sequencing and gene expression data from high-grade serous ovarian cancer patients, revealing that elevated expression of the glucocorticoid receptor gene was correlated with poor overall survival in ovarian cancer, regardless of BRCA1 and BRCA2 mutation status (Veneris et al., 2019). Our study observed that the expression level of NR3C1 was significantly lower in the BOTs group than in the group of BeOTs.

Currently, the molecular mechanisms underlying the involvement of NR3C1 in tumorigenesis and progression remain incompletely elucidated. Nandini Acharya et al. observed a progressive upregulation of glucocorticoid receptor (GR) expression and signaling from the naive state to dysfunctional CD8^+^ tumor-infiltrating lymphocytes (TILs). These glucocorticoids are locally produced by tumor-associated monocyte-macrophage cell lines, and the impact of GC on CD8^+^ T cells is contingent upon NR3C1. Conditional deletion of GR in CD8^+^ TILs significantly ameliorated this differentiation, decreased the expression of transcription factor TCF-1, and suppressed the dysfunctional phenotype, ultimately impeding tumor growth. The authors propose that endogenous steroid hormone signaling drives dysfunction in CD8^+^ TILs, with implications for cancer immunotherapy (Acharya et al., 2020). Amy R. Dwyer et al.'s research shows that cell damage, stress, and related factors in the tumor microenvironment can activate MAPK, causing phosphorylation of the glucocorticoid receptor at Ser134, which regulates the migration-related (NEDD9, CSF1, RUNX3) and metabolism-related (PDK4, PKG1, PFKFB4) gene sets to regulate the development and progression of triple-negative breast cancer. This discovery provides a potential new therapeutic target for TNBCs (Dwyer et al., 2023). NR3C1 is a potential downstream target of the NF-κB pathway, and silencing MDK (Midkine) by suppressing NF-κB activation and nuclear distribution reduces NR3C1 expression, thereby significantly inhibiting BC cell proliferation and migration (Zhang et al., 2022). The FKBP4/NR3C1/NRF2 signaling pathway has also been shown to be one of the pathways regulating BC cell autophagy and proliferation (Xiong et al., 2022). Minbo Yan et al. observed that when NR3C1 expression was silenced in renal cancer cells, lipid metabolism disorder, endoplasmic reticulum stress, and expression of mitotic genes were significantly enriched, which may be achieved through the ATF6-PINK1/BNIP3 pathway (Yan et al., 2023). Zhongbo Han et al.'s research found that microRNA-19b can target NR3C1 for downregulation, inhibiting cell apoptosis through the PI3K/AKT/mTOR pathway, thereby enhancing the resistance of colorectal cancer patients to oxaliplatin (Han et al., 2021). It can be seen that NR3C1 plays a role in cell regulation through multiple pathways and mechanisms. Our study only found that the role of NR3C1 in the development of BOTs may be achieved through regulating the expression of gamma delta T cells and CD4 memory resting cells, but the specific molecular mechanisms and pathways involved remain to be further elucidated by future relevant studies.

There are still several limitations to this study. The data sets used were solely obtained from the GEO database, and the sample size is relatively small. Furthermore, it should be noted that this study represents a bioinformatics analysis based on publicly available data. The diagnostic value and associated molecular mechanisms of NR3C1 as a clinical biomarker for BOTs still require validation through large-scale prospective studies in the future.

## Conclusion

In summary, by employing machine-learning techniques, we delved into the potential link between immunity and the development of BOTs. Our research revealed a significant association between the two. We finally determined that the NR3C1 gene affects the occurrence of BOTs through an immune-related pathway. This provides a strong basis for the early diagnosis and treatment of BOTs in the future.

## Data Availability

The original contributions presented in the study are included in the article/Supplementary Material, further inquiries can be directed to the corresponding authors.

## Supplementary material

SUPPLEMENTARY MATERIAL S1 A PCA plot showed the presence of batch effects and confirmed the comparability of the merged data.
