# Supplementary figures and images for: Identification of the key immune gene NR3C1 as a diagnostic biomarker in differentiating ovarian borderline tumors from benign tumors
Source: Front Cell Dev Biol. 2025 Sep 1;13:1602192. doi: 10.3389/fcell.2025.1602192 (PMC12434131; doi:10.3389/fcell.2025.1602192)

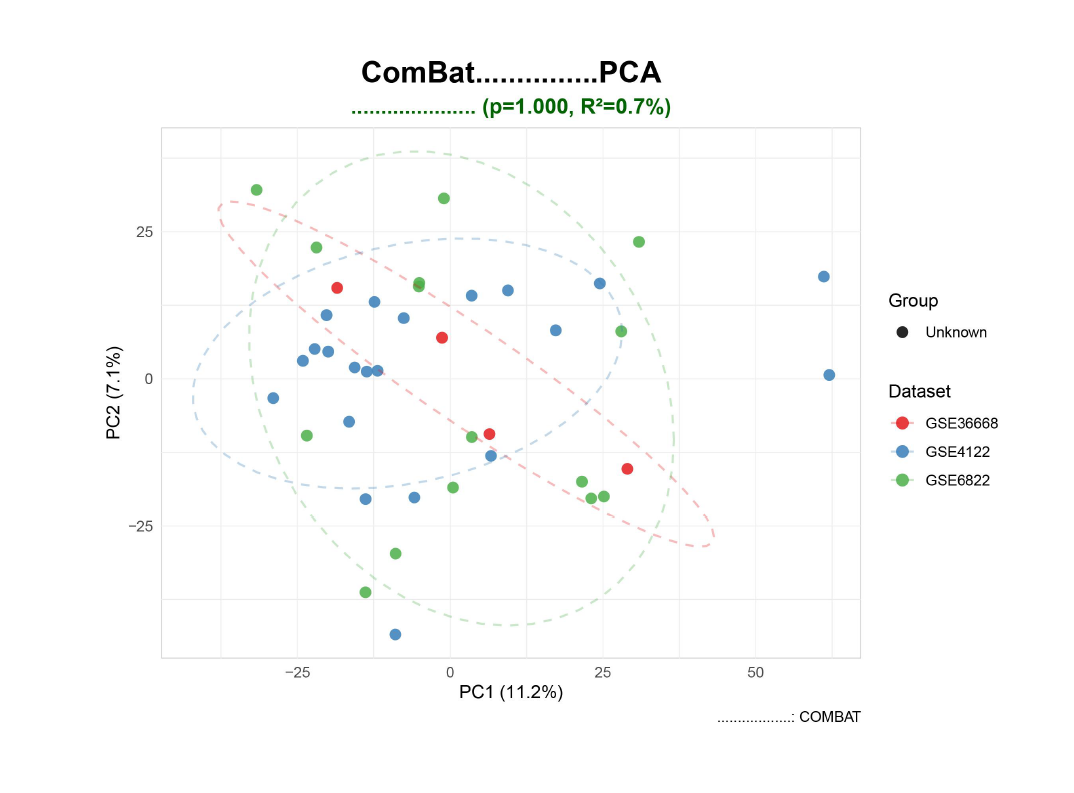

Supplement: Supplementary file 1 [file DataSheet1.zip › revised supplementary/Figure S1.tif]
